# Endoscopic management of multiple sessile serrated lesions in both the ileocecal region and the appendix cavity

**DOI:** 10.1055/a-2418-0499

**Published:** 2024-10-02

**Authors:** Jingjing Yao, Kunpeng Liu, Guangyao Zhao, Zhigang Wang, Xiao Wang, Jindong Fu

**Affiliations:** 1 549615 Department of Gastroenterology, Rizhao Peopleʼs Hospital, Rizhao, China; 2 549615 Department of General Surgery, Rizhao Peopleʼs Hospital, Rizhao, China; 3 549615 Department of Anesthesiology, Rizhao Peopleʼs Hospital, Rizhao, China; 4 549615 Department of Pathology, Rizhao Peopleʼs Hospital, Rizhao, China

A 54-year-old woman with no symptoms underwent a colonoscopy due to a family history of colon cancer. The procedure revealed a 2.0-cm laterally spreading tumor in the ileocecal region, adjacent to the appendiceal orifice ( Fig. 1 ). Abdominal computed tomography (CT) demonstrated a normal appendix. To further examine the appendix, a cholangioscope was utilized ( Fig. 2 ), which unveiled two areas of rough, granular mucosa within the appendix cavity, deemed abnormal ( Fig. 3 ). After obtaining informed consent, endoscopic removal of both the ileocecal lesion and the appendix was performed ( Video 1 ).

**Fig. 1:**
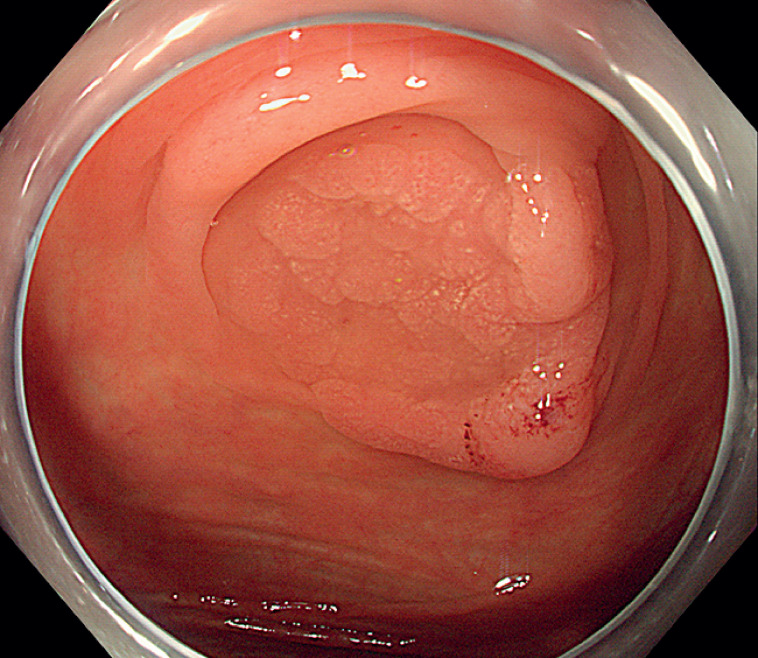
The colonoscopy revealed a 20-mm laterally spreading tumor in the ileocecal region.

**Fig. 2:**
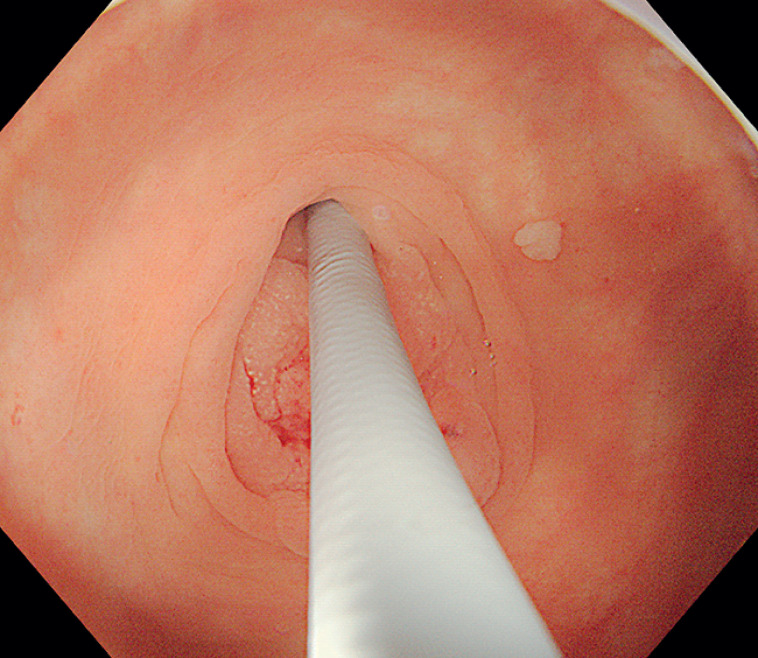
A cholangioscope was utilized to further examine the appendix.

**Fig. 3:**
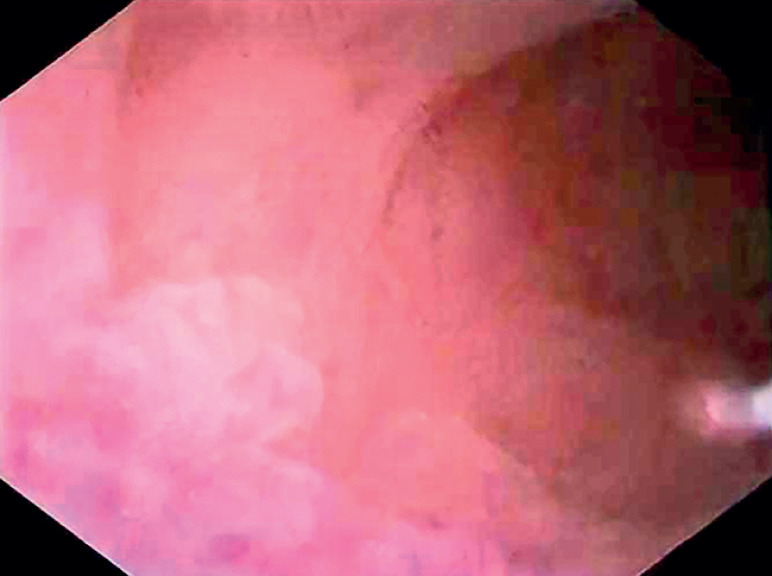
The rough, granular mucosa was unveiled within the appendix cavity.

Endoscopic removal of the ileocecal lesion and the appendix.Video 1

Following submucosal injection, the ileocecal lesion was excised entirely using a GoldKnife (Micro-Tech, Nanjing, China), employing the endoscopic submucosal dissection technique. Subsequently, an endoscopic full-thickness resection of the cecum tissue surrounding the appendiceal orifice was executed. An IT knife and GoldKnife were used to dissect the mesoappendix, proceeding along the appendix from its base. During the dissection, an elastic band was used to secure the appendix to the intestinal wall, facilitating traction. Once fully detached, the appendix was extracted from the intestinal lumen using a snare device ( Fig. 4 ). After thorough hemostasis, the wound was completely sealed using a StarClip (HCCD-0-195-M-C, Micro-Tech).

**Fig. 4:**
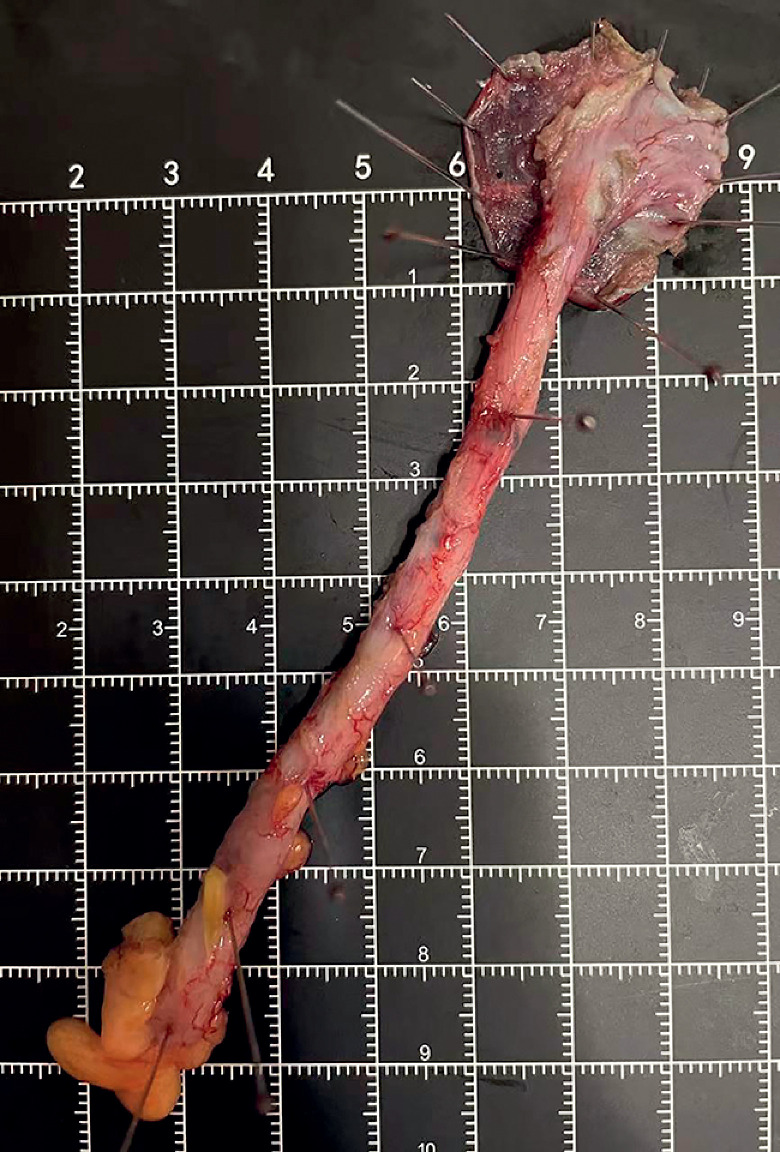
The removed appendix.

Postoperative histopathological analysis confirmed the presence of sessile serrated lesions (SSLs) in the ileocecal lesion and the rough areas within the appendix cavity, characterized by distorted serrated crypts, deep crypt serrations, and basal crypt dilation ( Fig. 5 ). The patient was kept fasting for 72 hours post-procedure and was administered antibiotic therapy. She experienced mild abdominal pain post-surgery but made a swift recovery and was discharged 5 days after the procedure

**Fig. 5:**
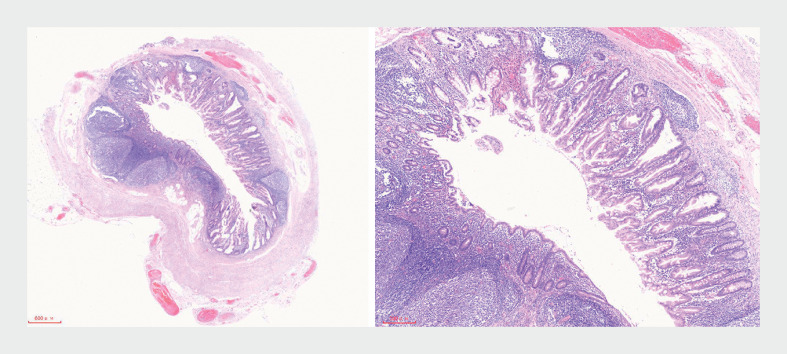
Pathology of the lesion within the appendix cavity.

Sessile serrated lesions are predominantly located on the right side of the colon and may extend to the appendix 1 . When confined entirely within the appendiceal lumen, these lesions are nearly undetectable by conventional colonoscopy. This case highlights the importance of considering the presence of SSLs in the appendix cavity when such lesions are identified in the colon.

Endoscopy_UCTN_Code_TTT_1AQ_2AD_3AF

## Notes

Endoscopy E-Videos https://eref.thieme.de/e-videos
*E-Videos* is an open access online section of the journal *Endoscopy* , reporting on interesting cases and new techniques in gastroenterological endoscopy. All papers include a high-quality video and are published with a Creative Commons CC-BY license. Endoscopy E-Videos qualify for HINARI discounts and waivers and eligibility is automatically checked during the submission process. We grant 100% waivers to articles whose corresponding authors are based in Group A countries and 50% waivers to those who are based in Group B countries as classified by Research4Life (see: https://www.research4life.org/access/eligibility/ ). This section has its own submission website at https://mc.manuscriptcentral.com/e-videos .
